# Controversies in Assessment, Diagnosis, and Treatment of Kratom Use Disorder

**DOI:** 10.1007/s11920-024-01524-1

**Published:** 2024-08-13

**Authors:** Kirsten E. Smith, David H. Epstein, Stephanie T. Weiss

**Affiliations:** 1 Department of Psychiatry and Behavioral Sciences, Johns Hopkins University School of Medicine, Baltimore, MD USA; 2 Translational Addiction Medicine Branch, Intramural Research Program, National Institute on Drug Abuse, National Institutes of Health, 251 Bayview Blvd., Baltimore, MD 21224 USA

**Keywords:** Kratom use disorder, Kratom addiction, *Mitragyna speciosa* addiction, Substance use disorder – other, DSM-5 diagnosis of substance use disorders

## Abstract

**Purpose of Review:**

We apply the Diagnostic and Statistical Manual of Mental Disorders Fifth Edition (DSM-5) criteria for substance use disorders (SUDs) to the herbal product kratom. Similarities and differences between kratom use disorder (KUD) and other SUDs are explored, along with assessment, diagnostic, and therapeutic recommendations for KUD.

**Recent Findings:**

Literature reports of “kratom addiction” or KUD rarely specify the criteria by which patients were diagnosed. Individuals meeting DSM-5 KUD criteria typically do so via tolerance and withdrawal, using more than intended, and craving, not functional or ​psychosocial disruption, which occur rarely. Most clinicians who use medication to treat patients with isolated KUD select buprenorphine formulations, although there are no controlled studies showing that buprenorphine is safe or efficacious in this patient population.

**Summary:**

Diagnosis and treatment decisions for KUD should be systematic. We propose an algorithm that takes into consideration whether KUD occurs with comorbid opioid use disorder.

## Introduction: Some Terminological Housekeeping for Substance Use Disorders (SUDs)

To discuss the addictive potential of any substance, it is first necessary to agree on a definition of *addiction*. In this paper, we use *addiction* synonymously with *substance use disorder* (SUD), as operationalized through eleven criteria in the *Diagnostic and Statistical Manual of Mental Disorders*, 5^th^ Revision (DSM-5) [1]. The DSM is a United States (US)-based nosology, but its SUD criteria are broadly consistent with those used for substance-related diagnoses in the leading international classification of diseases [2]. In both nosologies, addiction is broadly characterized as a pattern of substance use that occurs and persists despite detrimental net effects on patient well-being. These detriments may be medical, psychological, social, occupational, or some combination thereof. We emphasize *net* detriment because most drugs can have both desired and deleterious effects. Diagnosing an SUD requires determining that the harms of use outweigh the benefits for a specified time period, typically one year.

Accordingly, the DSM-5 criteria for SUDs characterize addiction in terms of its consequences. Any single DSM-5 criterion is neither necessary nor sufficient for diagnosis of an SUD. The criteria do not directly reflect frequency of use, and they are mostly silent on motivations for use, except for use to avoid withdrawal symptoms. There is nothing pathognomonic about drug use to self-medicate negative emotions, or to achieve euphoria (i.e., a recreational “high”). We know of only one scheme to incorporate drug use motivations into SUD criteria—specifically, a proposed diagnostic exemption for use of opioids to alleviate physical pain [3]. This proposal has not gained traction, and so SUD diagnosis remains driven largely by the net consequences of the person’s use.

In this paper, we apply the DSM-5 framework to define addiction to an increasingly used psychoactive botanical, *Mitragyna speciosa*, commonly referred to as “kratom.” We refer to kratom addiction as Kratom Use Disorder (KUD), a usage consistent with DSM-5 classifications. The DSM-5 SUD chapter names several substance-specific diagnoses, but follows them with an “other” category and these instructions: “When the substance is known, it should be reflected in the name of the disorder upon coding (e.g., nitrous oxide use disorder)” [1]. Thus, KUD, though not named in DSM-5, is a permissible DSM-5 diagnosis. Evaluation of a patient’s kratom use is complicated by the complex pharmacology of kratom leaves, the ever-growing number of kratom product formulations, and the frequent co-use of kratom with other substances. To date, there are few published clinical data on the characteristics of KUD. Here, we review relevant literature and provide recommendations for clinicians to assess, diagnose, and care for patients with KUD.

## Pitfalls of Conflating Kratom Physical Dependence with KUD/Addiction

Having chosen this DSM-5-based framework, which reflects the criteria used by US clinicians to evaluate addiction, we note a diagnostic pitfall relevant to kratom. With the shift from DSM-IV to DSM-5, the threshold number of criteria for an SUD diagnosis was reduced from three to two. That change reinstated the possibility of diagnosing an SUD solely based on the criteria of tolerance and withdrawal. The presence of either of those two criteria is traditionally called “physiological dependence” (DSM-IV) or “physical dependence.” With the DSM-5, the terminology was updated to “pharmacological criteria for dependence,” which avoids the problematic implication that some dependence is not physical.

Here, we use the term “physical dependence” because it remains standard in peer-reviewed literature, but we recommend that tolerance and withdrawal be discussed specifically instead. Each of these two criteria has two parts: the tolerance criterion can be met by a person endorsing using more drug to achieve the same effect, and/or feeling less effect from a given drug amount, while the withdrawal criterion can be either the experience of substance-specific withdrawal symptoms upon cessation, or continuing use to avoid such symptoms (negative reinforcement) [1]. By those two criteria, an SUD diagnosis would apply to many people who take prescribed psychiatric medications, because such medications often induce tolerance on initial titration and cannot be abruptly discontinued without withdrawal symptoms [4].

The DSM-5 provides a workaround in that tolerance and withdrawal are not counted toward an SUD diagnosis for medications taken as prescribed by a physician [1]. However, substances like kratom that cannot be prescribed by a physician cannot be similarly exempted. Thus, kratom consumers who meet tolerance and withdrawal criteria could be diagnosed with an SUD, while people with identical patterns/consequences of use for a prescribed drug, taken as directed, would not be diagnosed with an SUD. This inconsistency complicates comparisons of addiction liability for kratom versus prescribed drugs.

Another significant limitation of applying DSM-5 SUD criteria to kratom addiction is the uncertainty about whether the SUD category truly reflects one diagnostic entity. The DSM-5 workgroup, collapsing the older categories of “Dependence” and “Abuse” into the single category of SUD, argued that the 11 criteria appeared statistically unidimensional, representing one underlying construct [5]. That unidimensional view of SUDs is probably unrealistic, and the DSM-5 SUD criteria have been criticized for giving identical diagnoses to patients who may have none of the same symptoms [6]. While this heterogeneity may render the DSM-5 SUD criteria unhelpful for studies of etiology or physiopathology, the criteria do identify a category that is clinically and socially meaningful—the subset of patients having physical and/or psychosocial impairments and adverse consequences from substance use [7].

Finally, the DSM-5 subdivides its SUD criteria into four categories with no cited empirical support: impaired control, social problems, risky use, and the two “pharmacological criteria” [1]. A more convincing empirical subdivision is a factor analysis postdating the publication of the DSM-5, in which the unidimensional structure for alcohol use disorder (AUD) was shown to be mostly an artifact of using only one assessment question per criterion. With richer assessment, AUD appeared to reflect three dimensions: loss of control, withdrawal, and tolerance [8]. This finding implies that tolerance should likely not be lumped with withdrawal as if both reflected a single construct. We underscore this point because withdrawal (or use to avoid withdrawal) is one of the chief symptoms reported in KUD assessments to date.

## KUD: Prevalence and Typical Features

A DSM-5-based framework for KUD confers the possibility of comparing kratom with other substances using identical sets of diagnostic criteria. However, a disadvantage is that, to our knowledge, DSM-5-based criteria for KUD have been applied only to US populations. It is nonetheless important to comment on problematic kratom use in Southeast Asia, where kratom grows natively and is used within longstanding cultural traditions [9]. Cultural familiarity might be expected to protect against addiction, because open propagation of drug-related practices and prohibitions, with social learning about their possible benefits and harms, may constrain behavioral excesses [10]. Yet even in a Southeast Asian sample, more than half of kratom consumers scored medium or high on a proposed scale for “kratom dependence” [11]. This scale assessed several SUD-like constructs: preoccupation/​craving, impaired control, use despite harm, withdrawal, and tolerance [12]. However, a subsequent review concluded that the most common manifestations of kratom addiction in Southeast Asia were withdrawal symptoms [9]. That finding is consistent with self-reports from US consumers; some may not experience notable withdrawal symptoms upon discontinuation, but others do [13, 14]. When kratom withdrawal occurs, consumers usually characterize it as mild to moderate and specify symptoms such as kratom craving, low energy, fatigue, irritability, fatigue, anxiety, depressed mood, restless legs, difficulty sleeping gastrointestinal upset, cold and hot flashes, goosebumps, and muscle twitches [15–18]. To date there has been no laboratory-based human study of kratom withdrawal syndrome and no validation of a measure to assess it.

The first US-based survey to apply DSM-5-based KUD criteria to kratom-using adults was conducted in 2017 [19]. Among 2,798 respondents, 344 (12.3%) met the diagnostic threshold for past-year KUD; of these, 276 (80.2%) reported 2-3 criteria (mild KUD), 51 (14.8%) had 4-5 criteria (moderate KUD), and 17 (4.9%) had 6+ criteria (severe KUD) [19]. The authors did not specify how many of the mild KUD cases reported tolerance and withdrawal as their only criteria. A more recent survey of 2,061 respondents in 2023 found that 525 (25.5%) met past-year KUD criteria, most commonly due to tolerance (81.3%) and withdrawal (68.0%) [20]. Sampling differences preclude conclusions about whether the higher rate of cases in the second study represents a true increase.

In online surveys in 2020-2021, we identified a subset of adults who had ever used kratom among a broader sample, initially to represent a range of nonproblematic and problematic use of alcohol and other drugs. We found that, of 2,615 respondents, 289 (11.1%) had ever tried kratom, with 174 (60.2%) of these using kratom within the past year [21]. Kratom use differed from alcohol and tobacco use in a key respect: increases in frequency of alcohol or tobacco use were almost always perceived by respondents as changes for the worse, whereas increases in frequency of kratom use were not [22].

This suggested that for some respondents, kratom use was not perceived as a net detriment to functioning—a finding that was supported when we recontacted 129 respondents with any lifetime history of kratom use. Of the 122 actively using kratom, 79.8% reported that they still felt acute effects with each dose, but only 7% of those found the effects incompatible with daily obligations; most reported that kratom effects were helpful for productivity [23]. When asked if they conceptualized kratom as “addictive or habit-forming,” 24.8% of respondents endorsed this, though it is unclear which construct (“addictive” versus “habit-forming”) had greater support, or whether respondents conceptualized them differently.

In the 129 lifetime kratom users, KUD rates were 52.7% never, 17.8% remitted, and 29.5% past-year [23]. Of the latter, 14.0% met the diagnostic threshold by having only two KUD criteria. The most frequently met criteria (>30% of cases each) were using more than intended, tolerance, withdrawal, and craving. The least frequently met (<15% of cases each) were interference with obligations, giving up activities, use despite its causing social problems, and use when hazardous [24].

In 2022, we enrolled a national sample of current kratom consumers for 15 days of smartphone-based ecological momentary assessment (EMA) preceded by a baseline survey [25], with in-person assessments and interviews for a subsample [13, 26, 27]. In this separate sample of 357 daily or near-daily kratom users, the prevalence of KUD (66.7%) was considerably higher than in previous samples [25]. Again, however, the most frequently met criteria were withdrawal, tolerance, using more than intended, and craving. Reports of interference with activities or obligations, or use despite social problems, were rare, even in empirically defined clusters of participants who used kratom more frequently than others. EMA data suggested that the more frequent users had titrated their kratom use to meet their goals (such as self-treating symptoms of opioid use disorder, relieving pain, and increasing energy and productivity) rather than having lost control of their use [25]. Even among those who met KUD criteria, most reported that kratom use conferred benefits and helped them achieve their daily roles and obligations, which is noteworthy in that, distinct from many other SUDs including OUD, psychosocial functioning appears to remain intact among the plurality of kratom consumers, at least among those who participated in this research [25].

## Should a KUD Assessment be Approached Differently Than Other Substances?

In prior publications, we have cautiously compared daily kratom use to daily caffeine use, and KUD to caffeine addiction [26, 27]. Notably, a diagnosis of caffeine use disorder is forbidden by all DSM editions, although (or perhaps because) many caffeine users would likely meet SUD criteria, particularly per DSM-5 where only two criteria need be met [28]. This differential treatment of caffeine may be due to uncertainty about whether caffeine-related problems constitute a “clinically significant disorder” [29].

DSM-5 includes criteria to assess Caffeine Intoxication, Caffeine Withdrawal, Unspecified Caffeine-Related Disorder, and Other Caffeine-Induced Disorders (e.g., sleep and anxiety disorders). Such assessments may similarly be useful for other legal psychoactive substances like kratom for which the psychosocial consequences of use are buffered by cultural acceptance of the substance or its typical effects. Caffeine and kratom product formulations, which vary considerably, are widely available, and, apart from scattered state and local kratom prohibitions, are not scheduled under the Controlled Substances Act by the US Drug Enforcement Administration. Thus, the net negative consequences of kratom or caffeine use are socially, culturally, and legally distinct from those of illicit drug use, which confers greater consequential hazard and higher possible DSM-5 criteria endorsement. To date, many kratom products formulations are primarily consumed orally in leaf-based formulations, with highly concentrated kratom extract products seemingly consumed less frequently among regular kratom consumers [25]. This is perhaps similar to the way most US caffeine consumers are coffee drinkers, not daily consumers of concentrated caffeine energy shots.

Caffeine and kratom consumption patterns have other similarities, as well as significant differences. Like caffeine, kratom has acutely energizing effects in regular users, whereby a regular morning serving may improve focus and performance without notable impairment [26, 27]. Like caffeine dependence, KUD may manifest primarily as a realization that use has become a prerequisite to normal or optimal functioning, and the main driver of use becomes avoidance of withdrawal symptoms—though, for either substance, some perceived benefits may remain, and psychosocial impairment is minimal or absent [13].

*Unlike* caffeine, kratom: (1) has pharmacological effects beyond the alertness increase associated with adenosinergic antagonism [30], (2) is sometimes used for those other effects, which can include euphoria that some consumers describe as opioid-like [13], and (3) can lead to life-disrupting addiction manifestations in a small but nonnegligible proportion of consumers [13, 24, 25]. In addition, unlike caffeine, kratom is increasingly being sold in concentrated formulations not reflective of the whole-leaf kratom products that have been on the market in the US in the last two decades, and use of such concentrated extract products could increase the likelihood of unintended effects [31] or exposure to adulterants and contaminants [32]. The latter issue is partly separate from the issue of addictiveness, because toxicity or overdose can occur with any use of a substance (such as use by a first-time consumer who has acquired a product that is mislabeled or adulterated). There are few documented cases of fatalities, overdose, or toxicity that seem attributable to kratom alone, and causation in such cases remains speculative; for more on that topic, we refer interested readers to specific reviews [33–37].

## Clinical Assessment and Diagnostic Approaches to KUD with Recommendations for Clinicians

There are few published case reports in which KUD is assessed or diagnosed using a DSM-5-based approach [38]. Rather, most reports do not use clinical nosology or proffer any clear assessment or diagnostic methods [39]. In our 2023 systematic review of clinical case reports comprising 55 published cases [15], we found that only one patient was formally evaluated for KUD using the DSM-5 framework, with a diagnosis of “Other Substance Use Disorder, in Withdrawal”; SUD for kratom was not specified, and severity was unreported [40]. Similarly, a case report series using the term “KUD” provided no description of DSM-5-based assessment, diagnosis, or severity. Even in published cases where the authors state that DSM-5 criteria were used to diagnose patients with KUD, details of the assessment are typically not given [41, 42]. This is a concerning omission in the KUD literature given that, in many reported cases, patients were treated with a medication for opioid use disorder (MOUD) without a clear diagnosis or treatment rationale reported [43].

We have previously recommended that researchers and clinicians publishing kratom case reports should clearly describe their assessment and diagnostic criteria [15]. This is important for two reasons: first, to ensure that KUD assessment is performed systematically and consistently as for other SUDs, and second, to ensure that clinicians can fully evaluate the strength and applicability of published KUD cases to their own patient populations. Given the relevance of DSM-5 criteria for KUD assessment, we similarly advocate for addiction clinicians to systematically apply DSM-5 criteria when assessing and diagnosing KUD as they do with other SUDs. A description of the many well-validated DSM-5-based instruments for diagnosing SUDs is beyond the scope of this review [44], but we recommend that clinicians assessing patients with suspected KUD should consistently utilize one of these validated instruments and document the complete detailed results for each patient.

As part of the baseline workup for a patient with suspected KUD, we also recommend obtaining not just a urine drug screen immunoassay, but also confirmatory testing with chromatography-mass spectrometry that is able to detect (and ideally, also quantitate) one or more kratom alkaloids. Typically, such testing will detect the most abundant kratom alkaloid mitragynine, and sometimes also its more potently opioidergic metabolite 7-hydroxymitragynine. The importance of alkaloid-specific testing is that kratom products are largely unstandardized and unregulated, so their use carries risks of adulteration, contamination, or substitution with other substances [32]. With appropriate testing, both the clinician and the patient can be reassured that the patient with suspected KUD is actually taking kratom as believed, rather than some other substance or substance combination.

## Current Treatment Approaches to Comorbid KUD/OUD with Recommendations for Clinicians

Treatment of KUD, as with all SUDs, must be individualized for each patient based upon their specific preferences, characteristics, and comorbidities. To date, MOUD, specifically buprenorphine or buprenorphine-naloxone, has been the most frequent approach for treating kratom-related physical dependence or addiction in the US [38, 41, 42, 45–51]. In many of these cases, the affected patients were also reported to have a comorbid opioid use disorder (OUD) [42, 46, 47, 49, 50], so that MOUD was an obvious therapeutic approach despite the current lack of clinical trials specifically showing the safety or efficacy of MOUD for patients with KUD.

Although we found, in a small case series, that buprenorphine dosing for patients with comorbid KUD/OUD might correlate with kratom amount used [42], a subsequent larger case series did not replicate this finding [48]. We therefore recommend that buprenorphine dosing be individually titrated for KUD/OUD patients based upon their clinical responses. While we are not aware of any published cases describing comorbid KUD/OUD treated with methadone or naltrexone, these MOUD options could also be reasonably considered for select patients. We thus agree with previous authors that, in cases where a patient with KUD presents with comorbid OUD, use of MOUD (not only buprenorphine, but also naltrexone or methadone when deemed clinically appropriate) should be first-line treatment as it is for patients with OUD and other comorbid SUDs [52].

There is little specific information on whether comorbid KUD/OUD patients benefit from augmentation of MOUD with behavioral therapies like contingency management (CM) or psychotherapy. One small case series described the successful use of CM as an adjunct to buprenorphine in three patients with comorbid KUD/OUD [53]. Despite the sparseness of the published evidence, it is reasonable to offer CM or evidence-based therapies (e.g., cognitive-behavioral therapy, acceptance and commitment therapy, etc.) as MOUD adjuncts to any KUD/OUD patients who would like to use them together given the possible benefit and the low risk of harm from such therapy combinations. However, because the risk of relapse is so high for OUD patients in the absence of MOUD [54–56], we recommend against treating patients with comorbid KUD/OUD with non-MOUD therapies alone until or unless such therapies are studied in an evidence-based way and found to be efficacious as standalone therapies in this patient population.

## How Should KUD Treatment be Similar and Dissimilar to Treatment of Other SUDs? Recommendations for Clinicians Treating Patients Weith Isolated KUD

The question of how to treat patients with KUD and no underlying OUD is more controversial. It is common to see reports calling kratom alkaloids “opioids,” and there are ample preclinical data showing that the major kratom alkaloid mitragynine is a partial agonist at mu opioid receptors (MORs) [57], and the metabolite 7-hydroxymitragynine is a full agonist [58] (though it may not reach high concentrations in plasma, at least in mice [59]). Such evidence prompted former US Food and Drug Administration (FDA) commissioner Scott Gottlieb to declare that kratom was an opioid with no medical benefit [60], a position that was bolstered by the FDA’s own *in silico* studies of kratom alkaloid binding to opioid receptors [61]. The anecdotal evidence provided by human studies, patient report, and case reports regarding the opioid-like effects of kratom (and the opioid withdrawal-like effects of kratom cessation) should also be taken seriously [19, 30, 62–64].

However, we argue that even isolated KUD should be considered more akin to multiple SUDs, because kratom contains dozens of alkaloids, many of which have actions besides MOR binding. These include kappa and delta opioid antagonism [65], alpha-2 adrenergic agonism [57], 5-HT_1A_ agonism [66], and perhaps adenosinergic antagonism [30], along with a complex array of dopaminergic actions (some possibly antidopaminergic) [67–69]. Thus, patients with KUD will often present with features of OUD mixed with other SUDs, such as stimulant use disorder (StUD). This complication does not negate the importance of identifying and treating the OUD-like aspects of the patient’s KUD, but the clinician should not focus on that to the exclusion of its StUD-like and other features.

The published literature on patients with isolated KUD suggests that buprenorphine may be an effective therapy for them as well as for those with comorbid KUD/OUD [38, 41, 45, 51]. Perhaps reflecting the lack of proven therapeutic options for this population, a survey of US addiction physicians found that, among 19 respondents who reported caring for patients with KUD without comorbid OUD, buprenorphine was the most frequently selected therapy (17/19) [52]. However, there has been no systematic study of indications for use of buprenorphine to treat KUD in the absence of OUD, and there is limited guidance on the risk-benefit balance of initiating buprenorphine or other MOUD in this clinical situation. Complicating the picture is the fact that buprenorphine itself has been reported to cause adverse effects in at least one such patient [51]. Initiation of buprenorphine in KUD-only patients should only be done after careful consideration of the risks and benefits by the clinician and patient together, and with the fully informed consent of the patient regarding all possible treatment options.

The literature on other MOUD approaches to treat isolated KUD is even more limited. The previously mentioned survey of addiction physicians found that 3/19 reported using naltrexone, and only 1/19 reported using methadone [52]. One published case report briefly describes use of naltrexone maintenance for a patient with KUD, although no long-term follow-up data were provided [70]. Another case report describes initiating naltrexone in a patient with isolated KUD before switching the patient to buprenorphine due to the lack of efficacy of naltrexone [38]. Naltrexone, despite the lack of evidence for its efficacy in KUD, may be reasonable in some patients with isolated KUD, particularly since many clinicians and patients may feel more comfortable with this “opioid-free” approach for patients with no OUD diagnosis. There are also some reports of using methadone for subacute “drug substitution therapy” in kratom consumers in Malaysia [71]; to our knowledge, no US cases have been published using methadone that way. While methadone could be reasonable for treating some patients with a comorbid OUD, it should not be a first-line therapy for most isolated KUD patients, due to understandable concern about exposing opioid-naïve patients to the risks of a full opioid agonist, along with the known adverse effects of methadone [72].

The extent to which isolated KUD can be successfully treated with non-MOUD strategies such as CM or psychotherapy alone is unknown. In the above-mentioned survey of addiction physicians [52], seven of the 19 who had treated patients with isolated KUD reported using psychotherapy as an adjunct to MOUD, but another reported using psychotherapy alone. As in patients with comorbid KUD/OUD, use of CM or psychotherapy for isolated KUD is unlikely to cause harm despite being of unknown efficacy. Supportive pharmacotherapies for kratom withdrawal to treat presenting withdrawal symptoms, along with psychotherapy, should be considered for isolated KUD patients who are willing to use them, either alone or in combination with MOUD if use of MOUD is deemed clinically appropriate.

**Fig. 1:**
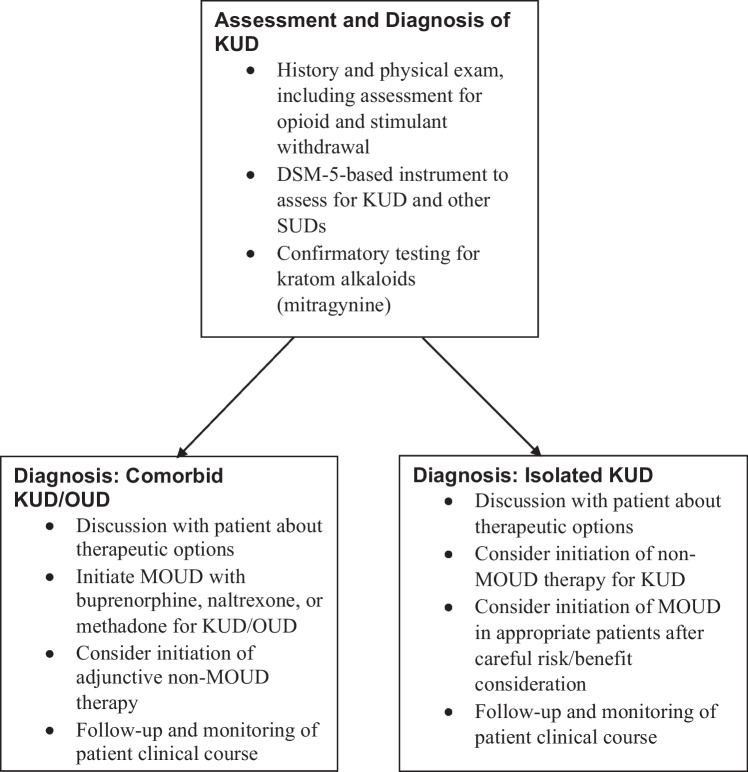
Algorithm of Considerations for the Assessment, Diagnosis, and Treatment of Patients with Suspected KUD

An algorithm of treatment considerations for KUD/OUD and isolated KUD can be found in Fig. 1.

## Conclusions

Currently there are no randomized controlled trials, or even long-term outcome studies, on treatment options for KUD. Until those kinds of systematic studies are published, clinicians need to rely on information from case reports and surveys, combined with their own judgment and experience. In this paper, we have tried to distill the available knowledge into an algorithm for decision-making.

Although KUD is not listed in DSM-5, it can be assessed and diagnosed using the same 11 criteria as other SUDs. While KUD shares many features with other SUDs, it has some distinguishing features. Specifically, many patients who meet criteria for a mild KUD diagnosis (equating to 2-3 DSM-5 criteria) will primarily do so because of tolerance and withdrawal rather than significant psychosocial impairment, although severe psychosocial impairment does occur in a minority of KUD patients. In addition, clinicians should be aware of the complex nature of KUD, both because kratom use often occurs in the context of polysubstance use, and also because the nature of kratom itself (with multiple alkaloids each having multiple actions) mimics the effects of multiple substances, with features of OUD but also of other SUDs, most notably StUD (or the technically undiagnosable caffeine use disorder). As with all SUDs, treatment options must be individualized based on patient characteristics and preferences, with extra care given to developing an appropriate treatment plan for patients who present with isolated KUD in the absence of OUD.

## Data Availability

No datasets were generated or analysed during the current study.
